# THE PREVENTION OF THE BEHAVIORAL AND PSYCHOLOGICAL SYMPTOMS IN DEMENTIA. HOW TO INVEST THE RESOURCES?

**DOI:** 10.1192/j.eurpsy.2023.1973

**Published:** 2023-07-19

**Authors:** A. Riolo, R. Zuttion

**Affiliations:** Azienda Sanitaria Universitaria Giuliano-Isontina, Trieste, Italy

## Abstract

**Introduction:**

The psychopathological observation of the behavioral and psychological symptoms in dementia (bpsd) and a study of the environment in which they arise, especially during a framework of mild cognitive impairment, must make us reflect on the consumption of resources for families and health or social services. The bpsd contribute 30% to the overall costs of dementia. In particular bpsd accelerate the institutionalization, temporary and definitive, of suffering elderly.

**Objectives:**

On the basis of some experiences, it is possible to use the health budget tool as part of the project budget, in the field of bpsd, moving from the practice of consumed cost to generated cost, that is the investment of resources that produces social value. The goal is to describe some concrete examples of this application, highlighting strenghts and weakness.

**Methods:**

The psychosocial interventions recommended to prevent the risk of onset of bpsd from a public health perspective are analyzed, in Italy and in other countries, in the light of the evolution of welfare theories and practices in community psychiatry.

**Results:**

The methodology of the health budget, in public health system, calls family members and users to a commitment of co-responsibility and administrators of financial resources in terms of accountability. The psychogeriatric elderly with mild cognitive impairment and bpsd, can also be a resource and not just a cost, if we consider the possible performance (dividend of longevity) and not the consumption of economic resources.

**Conclusions:**

A reconceptualization of the bpsd is necessary, which integrates the bio-medical dimension with psychosocial approach within the new welfare systems that must be rethought in function of the increase of the elderly population and respect to demographic trends. The onset of mild cognitive impairment and the reversibility of this pre-clinical occurrence should stimulate early recourse to appropriate psychosocial interventions to counter the social marginalization of many citizens.

**Disclosure of Interest:**

None Declared

